# Job vacancy at *Eurosurveillance* / ECDC

**DOI:** 10.2807/1560-7917.ES.2022.27.14.2204071

**Published:** 2022-04-07

**Authors:** Eurosurveillance editorial team

**Affiliations:** 1 European Centre for Disease Prevention and Control (ECDC), Stockholm, Sweden

The European Centre for Disease Prevention and Control (ECDC) invites applications for the following position:

 

**Scientific editor *Eurosurveillance***
The application deadline expires on 2 May 2022.

 

For more information, please visit the ECDC website: 

https://ecdc.europa.eu/en/work-us/vacancies?f%5B0%5D=deadline_date%3A1

